# Sustained Infiltration of Neutrophils Into the CNS Results in Increased Demyelination in a Viral-Induced Model of Multiple Sclerosis

**DOI:** 10.3389/fimmu.2022.931388

**Published:** 2022-09-29

**Authors:** Dominic D. Skinner, Amber R. Syage, Gema M. Olivarria, Colleen Stone, Bailey Hoglin, Thomas E. Lane

**Affiliations:** ^1^ Department of Pathology, Division of Microbiology and Immunology, School of Medicine, University of Utah, Salt Lake City, UT, United States; ^2^ Department of Neurobiology and Behavior, School of Biological Sciences, University of California Irvine, Irvine, CA, United States; ^3^ Department of Molecular Biology and Biochemistry, School of Biological Sciences, University of California Irvine, Irvine, CA, United States; ^4^ Center for Virus Research, University of California Irvine, Irvine, CA, United States

**Keywords:** neutrophils, neuroinflammation, chemokines, chemokine receptors, coronavirus

## Abstract

Intracranial inoculation of the neuroadapted JHM strain of mouse hepatitis virus (JHMV) into susceptible strains of mice results in acute encephalomyelitis followed by a cimmune-mediated demyelination similar to the human demyelinating disease multiple sclerosis (MS). JHMV infection of transgenic mice in which expression of the neutrophil chemoattractant chemokine CXCL1 is under the control of a tetracycline-inducible promoter active within GFAP-positive cells results in sustained neutrophil infiltration in the central nervous system (CNS) that correlates with an increase in spinal cord demyelination. We used single cell RNA sequencing (scRNAseq) and flow cytometry to characterize molecular and cellular changes within the CNS associated with increased demyelination in transgenic mice compared to control animals. These approaches revealed the presence of activated neutrophils as determined by expression of mRNA transcripts associated with neutrophil effector functions, including *CD63*, *MMP9*, *S100a8, S100a9*, and *ASPRV1*, as well as altered neutrophil morphology and protein expression. Collectively, these findings reveal insight into changes in the profile of neutrophils associated with increased white matter damage in mice persistently infected with a neurotropic coronavirus.

## Introduction

Intracranial viral infection of susceptible C57BL/6 mice with the neurotropic JHM strain of mouse hepatitis virus (JHMV) results in an acute encephalomyelitis associated with infection of astrocytes, microglia, and oligodendroglia with relative sparing of neurons (1–3). JHMV infection of the central nervous system (CNS) triggers an innate immune response resulting in upregulation of numerous pro-inflammatory cytokines and chemokines and rapid mobilization of the innate immune response characterized by recruitment of neutrophils and monocytes/macrophages to the CNS (4–6). Widespread dissemination of virus through the brain leads to secretion of T cell chemoattractants CCL5, CXCL9 and CXCL10 by resident cells of the CNS as well as by infiltrating innate immune cells (6–10). Secretion of these molecules aid in host defense by recruiting virus-specific CD4+ and CD8+ T cells to the CNS, which control viral replication through cytokine secretion and cytolytic activity (11–15). Despite this robust anti-viral cellular response, sterile immunity is not achieved, and viral antigen persists in white matter tracts, where demyelination is mediated, in part, by activated T-cells and macrophages (7, 16–18).

Neutrophils along with monocytes/macrophages comprise the predominant innate immune cell types mobilized early following CNS infection. ELR+ chemokines, CXCL1, CXCL2, and CXCL5, are expressed during both the acute and chronic stages of JHMV infection and are secreted by astrocytes in the CNS and function to recruit neutrophils *via* high-affinity binding to the chemokine receptor CXCR2 expressed on the surface of neutrophils (19). Neutrophils contribute to the breakdown of the blood-brain-barrier (BBB) early on during disease through the release of matrix metalloproteinases (MMP), which allow T-cells to enter the CNS and control viral replication. Blocking migration of neutrophils results in reduced T cell infiltration into the CNS that correlates with impaired control of viral replication and increased mortality (19). While neutrophils are critical to the defensive response, they exhibit potent defense mechanisms that can inadvertently cause extensive damage to surrounding tissues and exacerbate disease conditions. Upon activation, neutrophils exert anti-microbial activities and a variety of mechanisms that aid in controlling the spread of foreign pathogens and amplifying inflammation (20). These effector functions include phagocytosis, secretion of reactive oxygen species (ROS)/reactive nitrogen species (RNS) *via* degranulation, and release of neutrophil extracellular traps (NETs) (21–23). Additionally, neutrophils secrete a host of cytokines and chemokines that influence the recruitment of other immune cells to sites of inflammation (23). Neutrophils have been shown to play a role in the development of various autoimmune diseases including rheumatoid arthritis, systemic lupus erythematosus, and neuromyelitis optica (NMO) (24–27). An emerging role for neutrophils in MS has also been implicated (28–32), with elevated levels of CXCL1, CXCL5, and neutrophil elastase (NE) serum levels correlating with increases in the number and expansion of MRI lesions in MS patients (29).

Using a mouse model with tetracycline-inducible expression of CXCL1 from astrocytes, our lab demonstrated that sustained CXCL1 expression from the CNS results in increased neutrophil infiltration that correlates with increased clinical disease and demyelination in the JHMV model of viral-induced demyelination as well as in experimental autoimmune encephalomyelitis (EAE) the prototypic pre-clinical model of autoimmune-mediated demyelination. Conversely, blocking sustained neutrophil infiltration in both models ameliorated these effects, arguing that neutrophils can contribute to immune-mediated demyelination (33, 34). Mechanisms by which neutrophils may mediate white matter damage have yet to be well characterized. In the present study we used single cell RNA sequencing (scRNAseq) to show that CNS-infiltrating neutrophils have a distinct mRNA expression profile that impacts the immunological landscape during chronic disease. Neutrophils undergoing sustained CNS infiltration expressed transcripts *ASPRV1*, *CD63*, *MMP9*, *S100a8*, and *S100a9*, which have been associated with activation of neutrophil extravasation, chemotaxis, and increased effector functions. Additionally, we used flow cytometry to characterize associated changes in the morphology and surface marker expression of neutrophils. Collectively, these findings reveal multiple pathways by which neutrophils may contribute to white matter demyelination and offer new areas therapeutic targets to ameliorate demyelination.

## Materials and methods

### Mice and viral infection

pBI-CXCL1-rtTA double transgenic mice (C57BL/6, H-2^b^ background) were generated and bred as previously described (33, 34). In brief, pBI-CXCL1 transgenic mice were generated by the University of California, Irvine transgenic mouse facility through DNA microinjection of fertilized C57BL/6 eggs using the linearized pBI-CXCL1 construct. Founder transgenic (tg) mice were mated to wildtype C57BL/6 mice to identify F1 offspring containing the transgene. Hemizygous pBI-CXCL1 transgenics were crossed to hemizygous GFAP-rtTA*M2 mice (JAX), resulting in double transgenic (double-tg) mice (pBI-CXCL1-rtTA), single-tg (rtTA-GFAP or pBI-CXCL1) or wildtype. Doxycycline (50 mg/kg) administration *via* daily intraperitoneal (i.p.) injection for a period of 10 days to pBI-CXCL1-rtTA, but not either rtTA-GFAP or pBI-CXCL1 results in increased CXCL1 protein production within the CNS as measured by enzyme-linked immunosorbent assay (ELISA) and neutrophil accumulation in the CNS as determined by flow cytometry and immunohistochemical staining (33, 34). Double-tg mice and single-tg (male and female, 6-8 weeks) were injected intracranially (i.c.) in the right brain hemisphere with 250 plaque forming units (PFU) of JHMV strain suspended in 30μL of sterile Hanks balanced sterile solution (HBSS) and animals were euthanized at defined times post-infection (p.i.). Inoculation of mice with JHMV initially results in viral replication within the brain targeting glial cells with sparing of neurons and ultimately spreads to the spinal cord resulting in an immune-mediated demyelinating disease (2). To determine viral titers within brains, experimental animals were sacrificed at day 12 p.i. and trans-cardially perfused with 20mL of HBSS, brains isolated, homogenized and plaque assays were performed on DBT astrocytoma cell line as described previously (35). As a control, uninfected single-tg and double-tg mice were treated with Doxycycline (50mg/kg) *via* i.p. injection for 10 days and animals were sacrificed two days later to assess neutrophil accumulation within the spinal cords by flow analysis and the presence of demyelination *via* Luxol Fast Blue (LFB) and Hematoxylin/Eosin staining. Clinical disease severity was assessed using a 4-point scoring scale as previously described (33). All animal studies were reviewed and approved by both the University of California, Irvine and University of Utah Institutional Animal Care and Use Committee (IACUC).

### Cell isolation and flow cytometry

Flow cytometry was performed to identify inflammatory leukocytes infiltrating into the CNS using established protocols (36, 37). In brief, single cell suspensions were generated from tissue samples by grinding with frosted microscope slides. Immune cells were enriched *via* a 2-step Percoll cushion (90% and 63%) and cells were collected at the interface of the two Percoll layers. Before staining with fluorescent antibodies, isolated cells were incubated with anti-CD16/32 Fc block (BD Biosciences, San Jose, CA) at a 1:200 dilution. Cells were stained with fluorescently tagged anti-mouse IgG or Armenian hamster anti-mouse IgG Abs for the following cell surface markers: CD63, CD62L, CD45, Ly6G, CXCR4, and CD11b. A detailed list of antibodies used is provided in **Table 1**. Gating strategies for flow cytometric analysis were as follows: neutrophils (CD45+Ly6G+) and aged neutrophils (CD45+Ly6G+CXCR4^hi^CD62L^low^). Samples were analyzed using a BD LSR Fortessa X-20 flow cytometer or Amnis ImageStream Mark II imaging flow cytometer (Millipore-Sigma) and analyzed with FlowJo (Tree Star Inc.).

**Table 1:** Antibodies used for flow cytometry.

Flow Antibody	Clone	Cat #	Company
**CD63**	NVG-2	143904	BioLegend
**CD62L**	MEL-14	104435	BioLegend
**CD45**	30-F110	17-0451-82	eBioscience/ThermoFisher
**LY6G**	1A8	551460	BD Biosciences
**CXCR4**	2B11	565019	BD Biosciences
**CD11b**	M1/70	101212	BioLegend

### Histology

Experimental mice were euthanized according to IACUC guidelines at defined timepoints p.i. and perfused with 1X PBS. Spinal cords were removed, fixed overnight in 4% paraformaldehyde at 4°C, and separated into eight 1.5mm sections. Sections were cryoprotected in 30% sucrose for five days before embedding in O.C.T. (VWR, Radnor, PA, USA). Eight µM thick coronal sections were cut and stained with Luxol fast blue (LFB) and hematoxylin/eosin (H&E) and between 4-8 sections/mouse analyzed. In brief, for scoring of spinal cord section, areas of total white matter and demyelinated white matter were determined with Image J Software and demyelination was scored as a percentage of total demyelination from spinal cord sections analyzed (37).

### Single cell RNA sequencing

10x scRNAseq was performed as previously described (38, 39). Immune cells were isolated as described above from the spinal cord at day 12 p.i and stained with DAPI and APC conjugated anti-CD45 for 20 minutes on ice in 1X PBS containing 0.5% bovine serum albumin (BSA). Live CD45+ cells were enriched using a BD FACSAria flow sorter and washed once with 0.04% BSA. Samples were then processed for single cell RNA sequencing *via* the 10X Genomics platform performed at the Huntsman Cancer Institute High Throughput Genomics Shared Resource Core Facility at the University of Utah Health Science Center. RNA sequencing was performed *via* Illumina NovaSeq 6000 next generation sequencer. Sequencing data was processed using the 10X Genomics CellRanger pipeline and analyzed using the Seurat R package. Gene expression signatures defining cell clusters were analyzed from double-tg and single-tg controls at day 12 p.i in spinal cord. Cells from each aggregated sample dataset were clustered into corresponding immune cell populations by a shared nearest neighbor modularity optimization-based clustering algorithm using the Seurat package. The resulting clusters were defined using an immune-cell scoring algorithm that compares the gene signatures of each cluster in the experimental dataset with the microarray data available in the Immunological Genome Project Database (40, 41). Expression levels and distribution of population-specific immune cell markers were then analyzed to further refine the identified clusters and expose any subpopulations that should be separated as independent clusters. Once the clusters were established and identified, plots were generated using Seurat, ggpubr and fgsea R packages.

### ELISA

To determine tissue protein level, tissue was homogenized in RIPA buffer, centrifuged, and supernatant aliquoted. Protein assays were run using CXCL1, NGAL, Neutrophil Elastase, S100a8, S100a9 DuoSet sandwich ELISA kit (R&D Systems, Minneapolis, MN) following manufacturer specifications.

### Statistical analysis

For flow cytometry analysis unpaired Student’s *t* test was used to determine significance and a *p* value of < 0.05 was considered statistically significant. GraphPad Prism was used to perform statistical analyses. Data for each experiment is presented as mean ± standard error of mean (SEM). Wilcoxon test was used for analyzing gene expression in scRNASeq clusters and the resulting *p* values were corrected for multiple comparisons by Holm-Sidak method and a *p* value of < 0.05 was considered statistically significant.

## Results

### JHMV infection of CXCL1 transgenic mice

**Figure 1:**
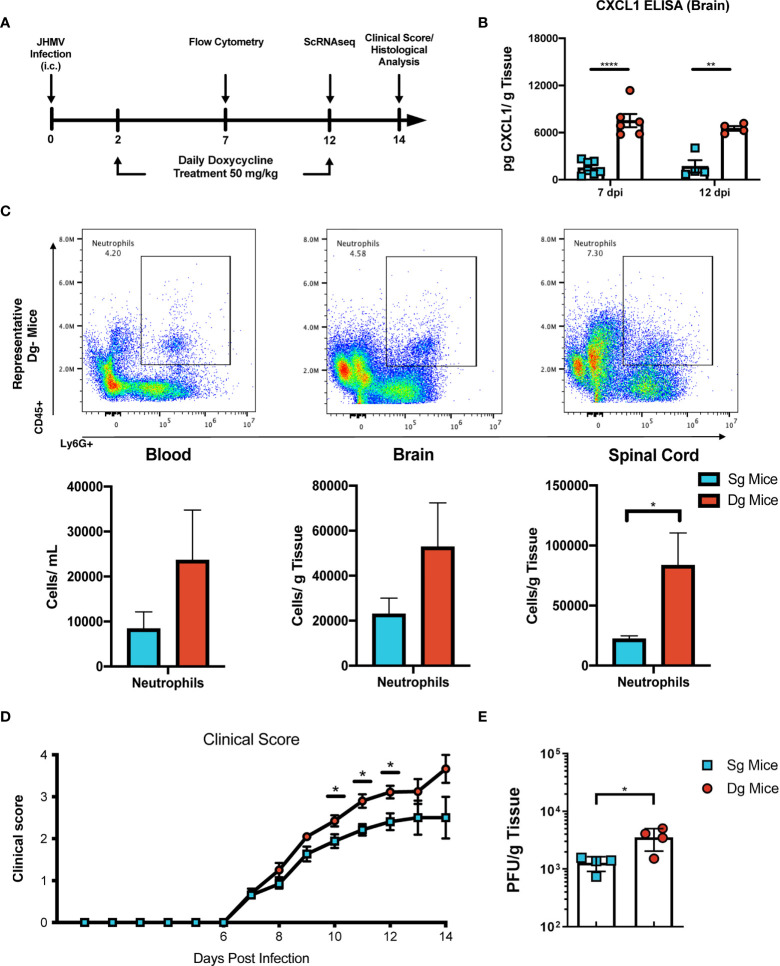
JHMV-infection of double-tg mice results in worsened clinical disease following doxycycline-induced CXCL1 expression. **(A)** Schematic overview showing experimental paradigm. Single-tg and double-tg mice were infected intracranially (i.c) with 250 PFU of JHMV and treated daily with doxycycline (50 mg/kg) *via* i.p. injection from 2-12 days p.i. Tissue was collected and analyzed at defined times. **(B)** ELISA was performed using supernatants of homogenized infected brain tissue at days 7 (n = 6-7 mice per group) and 12 (n = 4 mice per group) p.i.; double-tg mice showed elevated levels of CXCL1 protein at both timepoints following doxycycline treatment compared to single-tg control mice. Data is derived from a minimum of 2 independent experiments per timepoint. **(C)** Flow cytometry dot plots showing increased neutrophil (CD45+Ly6G+) accumulation in the blood, brain and spinal cords of double-tg mice compared to single-tg control. Data is derived from 2 independent experiments with a minimum of 5 mice used per tissue type. **(D)** Representative clinical disease showing JHMV-infected double-tg (n = 5) with increased (p < 0.05) clinical disease severity compared to infected single-tg mice (n = 6) at defined times p.i. **(E)** Viral titers are increased (p < 0.05) within the brains of JHMV-infected double-tg mice (n = 4) compared to infected single-tg mice (n = 4) at day 12 p.i. at day 12 p.i. *p< 0.05, **p< 0.01, ****p< 0.0001.

**Figure 2:**
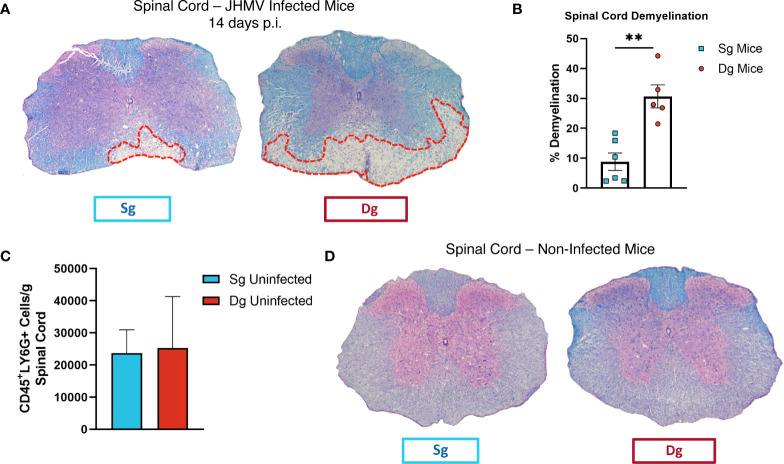
Demyelination is increased in doxycycline-treated double-tg infected with JHMV. **(A)** Representative H&E/LFB-stained spinal cord sections from doxycycline-treated double-tg and single-tg mice infected with JHMV at day 14 p.i. reveals increased spinal cord demyelination (red dashed line) in double-tg mice compared to single-tg mice. **(B)** Quantification of demyelination severity indicates white matter damage is significantly (p < 0.01) more severe in infected double-tg mice (n = 5) compared to single tg mice (n = 6); data is derived from 2 independent experiments. **p< 0.01. **(C)** Flow cytometric analysis of neutrophils present in the spinal cords of uninfected doxycycline-treated single-tg mice (n = 3) and double-tg mice (n = 3) shows no significant difference in neutrophil numbers between experimental groups. **(D)** Representative H&E/LFB-stained spinal cord sections from uninfected doxycycline-treated single-tg mice (n = 4) and double-tg (n = 4) reveals no evidence of demyelination.

To better understand mechanisms by which neutrophils contribute to demyelination, doxycycline-inducible pBI-CXCL1-rtTA double transgenic mice (double-tg mice) and control rtTA-GFAP single transgenic mice (single-tg mice) were inoculated intracranially (i.c.) with JHMV, subsequently treated with doxycycline to induce CXCL1 expression within the CNS and sacrificed at defined times p.i. (**Figures 1A, B**). Our previous studies on these animals show that CXCL1 expression is restricted to GFAP-positive astrocytes and is not expressed by other CNS cells (33, 34). Flow cytometric analysis at day 7 p.i revealed a significant (p<0.05) increase in neutrophils (CD45+Ly6G+) in the spinal cord of JHMV-infected double-tg mice compared to infected single-tg mice (**Figure 1C**). In addition, neutrophil levels were increased in both blood and brain of JHMV-infected double-tg mice compared to infected single-tg mice although these differences were not significant (**Figure 1C**). Consistent with our previous studies, JHMV-infected double-tg mice exhibited more severe clinical disease compared to JHMV-infected single-tg control mice out to day 14 p.i (**Figure 1D**) and this was associated with an increase (p<0.05) in brain viral titers (**Figure 1E**). In addition, the severity of spinal cord demyelination was increased (p<0.01) in infected double-tg mice compared to single-tg mice (**Figures 2A, B**). We next tested whether sustained neutrophil infiltration into the CNS and demyelination occurs in the absence of JHMV infection of doxycycline-treated double-tg mice. Our findings indicate that there is no significant difference in the presence of neutrophils in the CNS of uninfected single-tg versus double-tg mice (**Figure 2C**). Examination of spinal cords from uninfected mice revealed no evidence of either inflammation in spinal cord white matter tracts or demyelination in either uninfected experimental group (**Figure 2D**). These findings indicate that JHMV infection of the CNS enhances neutrophil accumulation and demyelination in doxycycline-treated double-tg mice.

### Single cell RNA sequencing of CD45+ cells revealed increases in T cell and microglia subsets in JHMV-infected double-tg mice

**Figure 3:**
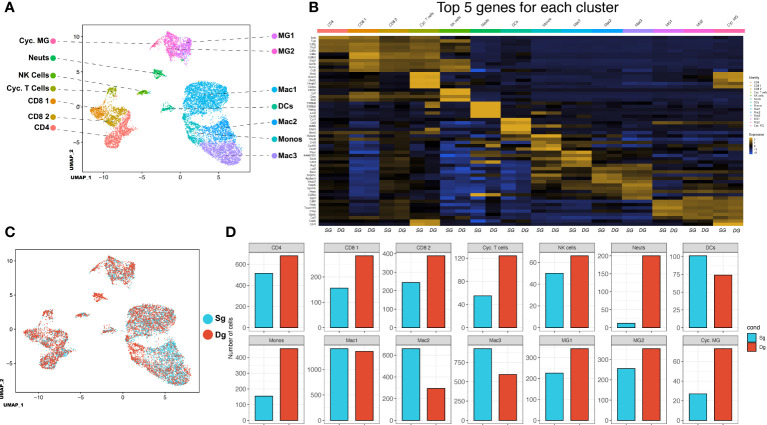
scRNAseq of CD45+ cells isolated from spinal cords of JHMV-infected mice.**(A)** Uniform Manifold Approximation and Projection (UMAP) plot of scRNASeq data revealing 14 distinct cell clusters (aggregate data from JHMV-infected double-tg (n = 4) and single-tg mice (n = 4) treated with doxycycline at 12 days p.i. **(B)** Heat map showing the top 5 differentially expressed genes with each cluster. Columns show each cluster, with sub columns representing single-tg and double-tg mice; rows specify genes. **(C)** UMAP plot displaying an overlay of single-tg (blue) and double-tg (red) scRNASeq data. **(D)** Number of cells per cluster comparing single-tg (blue) and double-tg (red) scRNASeq data from spinal cord at 12 days p.i.

Our findings indicate that elevated levels of CXCL1 protein and accumulation of neutrophils within the CNS associates with increased demyelination and disease severity. We employed 10x scRNAseq technology to characterize cellular and molecular changes in the immunologic landscape at peak disease in JHMV-infected double-tg mice as compared to infected single-tg control mice. JHMV-infected double-tg and single-tg mice were treated with doxycycline starting on day 2 p.i. for 10 days p.i. and sacrificed at day 12 p.i. at which point live cells were isolated from spinal cords and sorted based on CD45+ expression and processed using the 10x Genomics scRNAseq platform (38, 39). We aggregated data taken from both double-tg experimental mice and single-tg control mice and performed unbiased clustering analysis based on similarity of gene expression signatures using the Seurat single cell genomics R package and this approach revealed 14 distinct cell clusters (**Figure 3A**). In order to verify the algorithm-assisted identification of cell clusters, we examined expression of known cellular markers in our dataset and expression of these markers corresponded with the respective identities of the distinct clusters (**Figure 3B**). We next analyzed differences in the frequency of different CD45+ cell types between JHMV-infected double-tg and single-tg mice to better understand differences in gene expression profiles in immune cell subsets between experimental groups of mice. When compared side by side, differences in immune cell infiltration emerged (**Figure 3C**). There was an overall increase in CD4+ and CD8+ T cells as well as microglia in JHMV-infected double-tg experimental mice compared to infected single-tg mice. The monocyte population was also increased in infected double-tg mice, yet macrophage populations were reduced as compared to single-tg mice. Importantly, there was an increase in neutrophils in double-tg experimental mice compared to single-tg control mice, which was consistent with our previous studies (**Figure 3D**) (33, 34).

### Neutrophils express transcripts associated with demyelination

**Figure 4:**
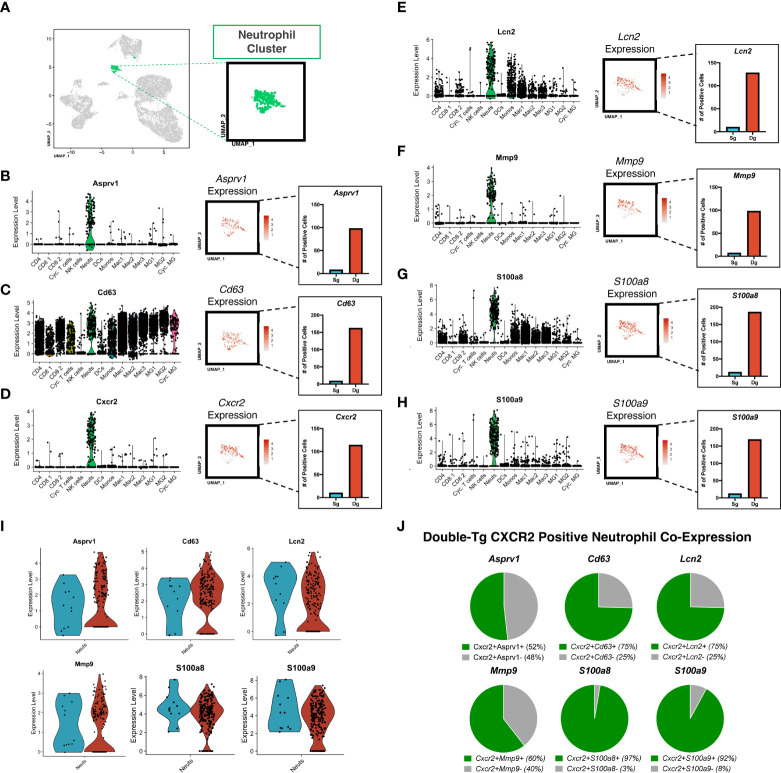
Neutrophils isolated from the spinal cord express genes associated with chemotaxis and demyelination. **(A)** UMAP plot showing highlighted (green) neutrophil cluster from JHMV-infected single-tg (n = 4) and double-tg (n = 4) mice. **(B–H)** Violin plots show expression of *Asprv1*, *Cd63*, *Cxcr2 Lcn2 (NGAL), Mmp-9*, *S100a8, S100a9* primarily within neutrophil clusters; black dots represent individual cells. Inset UMAP plots show the expression level of each gene within the neutrophil cluster. Bar graphs show the number of positive neutrophils for each respective gene for double-tg (red) and single-tg (blue) datasets. **(I)** Violin plots demonstrating that neutrophils from both JHMV-infected single-tg and double-tg mice express *Asprv1, Cd63, Cxcr2, Lcn2 (NGAL), Mmp-9, S100a8*, and *S100a9* transcripts. **(J)** Pie charts show the frequency of *Cxcr2+* neutrophils from the double-tg mice that express *Asprv1*, *Cd63*, *Lcn2 (NGAL), Mmp-9*, *S100a8, S100a9*.

Aggregate analysis of neutrophils isolated from the spinal cords of JHMV-infected single-tg and double-tg mice show a discrete clustering pattern (**Figure 4A**). Within this neutrophil cluster there are transcripts expressed associated with neutrophil activation and chemotaxis including *Asprv1* (aspartic retroviral-like protease 1), *Cd63*, *Cxcr2*, *Lcn2 (NGAL), Mmp-9*, *S100a8, S100a9* (**Figures 4B–H**). *Asprv1* expression has been shown to be restricted to extravasated ICAM1+ neutrophils within the immune system and to promote chronic inflammation in EAE (42). Indeed, expression of these molecules, with the exception of *Cd63*, is enriched almost exclusively in CNS inflammatory neutrophils in both JHMV-infected single-tg and double-tg mice (**Figures 4B–H**). Expression of *Cd63*, a marker expressed on the surface of neutrophils following activation and degranulation, was also increased in infected double-tg mice compared to the single-tg mice (43) (**Figure 4C**). *Cxcr2* expression was enriched as well, supporting prior research from our laboratory that CXCR2 is important for neutrophil trafficking into the CNS following JHMV infection (19, 33). Moreover, there were also more *Cxcr2*-positive neutrophils in the infected double-tg sample compared to infected single-tg controls (**Figure 4D** **)**. Similarly, *Lcn2 (NGAL)* expression, which has been implicated in amplification of neutrophil inflammatory signaling and recruitment (44), was also enriched in infected double-tg mice when compared to infected single-tg controls (**Figure 4E**). Additionally, we found that transcripts for the metalloproteinase MMP-9, an enzyme involved in BBB breakdown and pro-inflammatory signaling and the inflammatory signal-amplifying alarmins *S100a8* and S100a9, were highly enriched in neutrophils from double-tg mice compared to the single-tg controls (**Figures 4F–H**). Neutrophils from JHMV-infected single-tg and double-tg mice express similar levels of transcripts encoding *Asprv1*, *Cd63*, *Lcn2 (NGAL), Mmp-9*, *S100a8*, and *S100a9* transcripts (**Figure 4I**). It is important to note that neutrophils from both infected single-tg and double-tg express these specific transcripts and expression is neither specific nor enhanced in double-tg neutrophils. Rather, transcripts levels are increased due to the overall increased numbers of neutrophils present in the spinal cords of double-tg mice compared to single-tg mice. When looking at expression profiles of disease-associated transcripts in neutrophils isolated from the spinal cords of JHMV-infected double-tg mice in which demyelination is increased, the majority of these transcripts are enriched in *Cxcr2+* neutrophils (**Figure 4J**).

### Altered expression of T cell chemoattractant chemokines in the spinal cords of JHMV-infected double-tg mice

**Figure 5:**
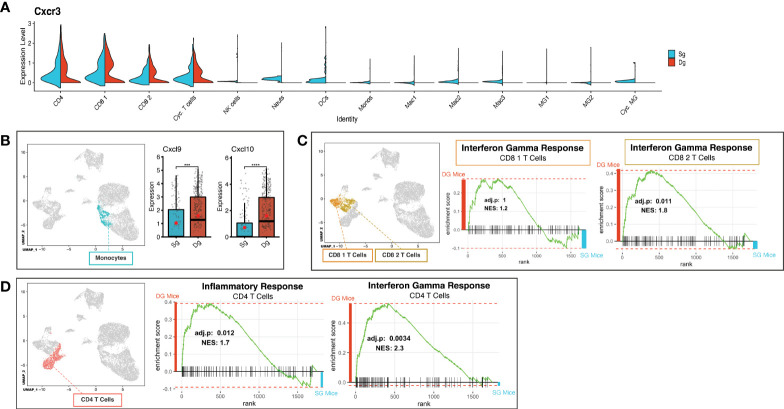
Neutrophil accumulation alters expression of T cell attractant chemokines and T cell activation state. **(A)** Split violin plot of scRNAseq data showing *Cxcr3* expression level (y-axis) compared by cluster (x-axis) with width of plot signifying frequency of *Cxcr3* expressing cells. *Cxcr3* expression relegated to T cell cluster subsets (CD4, CD8 1, CD8 2, Cyc. T Cells) in both single-tg (blue) and double-tg (red) mouse datasets. **(B)** Expression levels of chemokines *Cxcl10* and *Cxcl9* in monocytes are shown for single-tg (blue) and double-tg (red) mouse datasets. **(C)** Gene set enrichment analysis (GSEA) for IFN-γ responses in CD8 1 T cell and CD8 2 T cell clusters from spinal cord of single-tg (n = 4) and double-tg mice (n = 4) at day12 p.i. Responses to IFN-γ were enriched in both populations of double-tg CD8 T cells compared to control single-tg mice. **(D)** GSEA plot for inflammatory response and IFN-γ response in CD4 T cells. Responses to both inflammatory response and IFN-γ response was enriched in the CD4 T cell population of double-tg mice compared to control single-tg mice. The GSEA plots correspond to the designated Hallmark Inflammatory Response and Hallmark Interferon Gamma Response gene sets established in the Molecular Signatures Database (MSigDB) (45, 46). ***p < 0.001; ****p < 0.0001.

Demyelination in JHMV-infected mice is amplified by inflammatory activated T cells and macrophages (7, 16). To better understand how increased neutrophil infiltration of the CNS influences changes in T cells, we further probed the T cell cluster subsets *via* scRNAseq analysis to identify characteristics that might contribute to worsened disease outcome. We determined that expression of transcripts encoding the chemokine receptor CXCR3 was localized to T cell subsets (CD4, CD8 1, CD8 2, Cyc. T Cells) in both JHMV-infected double-tg mice and single-tg mice (**Figure 5A**). There was a significant increase in transcripts encoding the CXCR3 ligands CXCL9 (p<0.001) and CXCL10 (p<0.0001) in monocytes of double-tg mice compared to single-tg mice (**Figure 5B**) consistent with results from previous studies (39). Increased expression of *Cxcl9* and *Cxcl10* within the spinal cords of infected double-tg mice most likely accounts for the trending increase in T cell subsets, as these have been previously demonstrated to have important roles in attracting activated CD4+ and CD8+ T cell subsets into the CNS of JHMV-infected mice (10, 11, 15, 16, 47). GSEA analysis revealed both CD8+ T cell subsets exhibited enriched IFN-γ response in double-tg experimental mice compared to single-tg control mice (**Figure 5C**). Additionally, using GSEA analysis the CD4+ subset showed enriched inflammatory response genes and IFN-γ response genes in double-tg experimental mice compared to single-tg control mice (**Figure 5D**).

### Neutrophils from JHMV-infected double-tg mice exhibit an activated state within the CNS tissue

**Figure 6:**
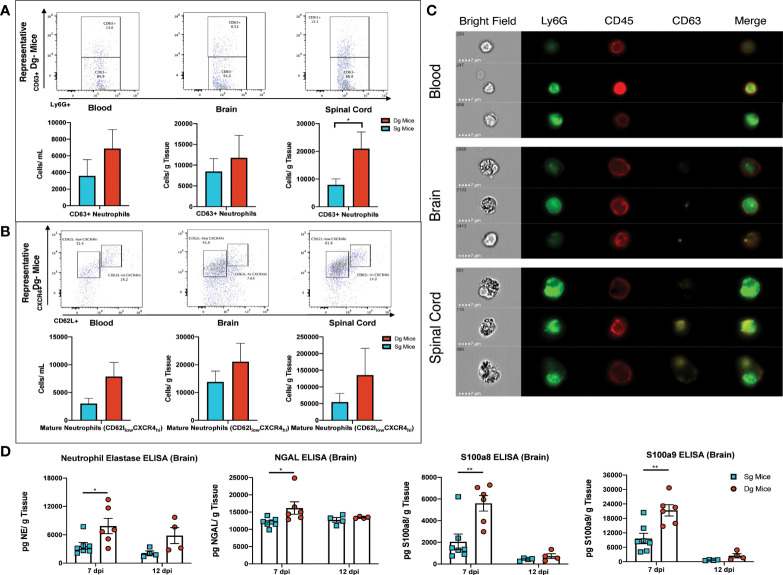
Neutrophils from JHMV-infected double-tg mice exhibit increased activation state **(A)** Representative flow plots reveal CD63+ neutrophils in the blood, brain, and spinal cord of JHMV-infected double-tg mice (n = 5) compared to infected single-tg (n = 7) control at day 12 p.i. Numbers of neutrophils in the spinal cord of infected double-tg mice are significantly (p < 0.05) increased compared to infected single-tg mice. **(B)** Representative flow plots showing mature neutrophils (CD45+Ly6g+CXCR4^hi^CD62L^low^) in blood, brain and spinal cords of JHMV-infected double-tg mice (n = 5) and infected single-tg mice (n = 7). **(C)** Representative ImageStream flow cytometry on neutrophils (CD45+Ly6G+) isolated from blood, brain and spinal cord obtained of JHMV-infected double-tg mice reveals enhanced CD63 expression in spinal cord-associated neutrophils. In addition, neutrophils from brains and spinal cords exhibited increased granularity compared to neutrophils obtained from the blood. For data in panels **(A–C)**, n = 5-7 mice per tissue type per group; 2 experiments. Representative flow plots are shown in panels **(A, B)**. **(D)** JHMV-infected double-tg and single-tg mice were sacrificed at days 7 (n = 6-7 mice per group) and 12 p.i. (n = 4 mice per group) and levels of Neutrophil Elastase, NGAL, S100a8, and S100a9 determined by ELISA (3 independent experiments). *p< 0.05, **p< 0.01.

Upon extravasation and infiltration of parenchymal tissue, neutrophils alter expression levels of surface markers that denote activation of their effector functions and exhibit an aged phenotype that is associated with greater degranulation, oxidative burst and delayed apoptosis (48, 49). Flow cytometry surface staining for the degranulation marker CD63 in conjunction with neutrophil markers (CD45+Ly6G+CD63+), showed CD63+ neutrophils in the brain and blood of double-tg mice and a significant (p<0.05) increase of CD63+ neutrophils in the spinal cords of these mice at day 12 p.i. (**Figure 6A**). Although numbers of these cells were increased in both the blood and brain of infected double-tg mice compared to single-tg animals, these differences were not significant (**Figure 6A**). In addition, there were increased numbers of mature neutrophils (CD45+Ly6G+ CXCR4^hi^CD62L^low^) in the blood, brain and spinal cords of JHMV-infected double-tg mice compared to infected single-tg control although these differences were not significant (**Figure 6B**). Examination of neutrophil morphology *via* ImageStream flow cytometry revealed a noticeable increase in the granularity of neutrophils isolated from the spinal cord and the brains of JHMV-infected double-tg mice compared to blood and this correlated with increased expression of CD63 around the outer cell membrane (**Figure 6C**). To compare expression of proteins associated with neutrophil activation and to verify protein expression of transcripts highlighted by scRNAseq, brains were isolated at days 7 and 12 p.i. from JHMV-infected single-tg control mice and double-tg mice and assayed *via* ELISA. Double-tg mice displayed increased (p<0.05) expression of neutrophil gelatinase-associated lipocalin (NGAL) at day 7 p.i. and, in correlation with scRNAseq results, increased NE, an enzyme involved in degranulation and the NET release pathway (**Figure 6D**). Lastly, there was increased (p<0.01) protein expression of S100a8 and S100a9 in the brains of JHMV-infected Dox-treated double transgenic mice compared to infected single transgenic mice at day 7 p.i. (**Figure 6D**).

## Discussion

Previous studies from our laboratory have demonstrated an important role for neutrophils in contributing to effective host response following JHMV infection of the CNS by increasing the permeability of the BBB and allowing CNS-infiltration of virus-specific T cells (19, 50). Our work has also demonstrated that under chronic neutrophil stimulation, sustained neutrophil infiltration of the CNS can lead to increased tissue damage and demyelination. Neutrophils have been shown to be important contributors to demyelination in several mouse models of MS (51–54). We have developed transgenic mice in which expression of the neutrophil chemoattractant, chemokine CXCL1, is under the control of a tetracycline-inducible promoter that drives the expression of this transgene within astrocytes. Using this transgenic mouse model, we were able to show that chronic CXCL1 expression from the CNS leads to sustained neutrophil infiltration which correlates with exacerbated spinal cord demyelination and clinical disease in both the EAE and JHMV models of demyelination (33, 34). Additionally, we demonstrated that in the absence of disease-induced neuroinflammation, there is low-level accumulation of neutrophils within the spinal cords of doxycycline-treated double-tg mice yet no evidence of immune cells infiltrating white matter tracts or demyelination, arguing that JHMV infection enhances neutrophil accumulation within the CNS. This difference underscores that JHMV infection results in an ~40-fold increase in CXCL1 protein levels and potentially amplifies the neutrophil response in the CNS of doxycycline-treated double-tg mice compared to naïve double-tg mice (34).

In the present study, we found that increased clinical disease severity in JHMV-infected double-tg mice was associated with increased neutrophil accumulation in the spinal cords and increased demyelination. Neutrophils secrete inflammatory signals and contribute to orchestrating the innate and adaptive immune responses. Activated neutrophils utilize positive feedback loops *via* autocrine and paracrine signaling to stimulate further neutrophil recruitment through chemoattractants such as CXCL1, CXCL2, LTB4 and cytokines like MPO, NGAL (LCN2), MMP-9, S100A8, and S100A9, respectively (55). Our data indicates an increased presence of many of these molecules, including MPO, NGAL, MMP-9, S100A8 and S100A9, in correlation with an increased number of neutrophils in the spinal cords of JHMV-infected double-tg mice, which suggests these as potential drivers of further neutrophil recruitment to sites of infection-induced neuroinflammation. Evidence from our scRNAseq analysis leads us to believe that the increased transcript levels for these genes is not selectively enriched in neutrophils from double-tg mice compared to single-tg mice. Rather, that the increased transcript levels simply reflect the overall increase in numbers of neutrophils within the spinal cords of infected double-tg mice responding to sustained CXCL1 expression. Alarmins S100A8 and S100A9 are calcium binding proinflammatory proteins that are members of the S100 family and most often exist in the form of heterodimers due to instability of their homodimer structure (56). Under high concentrations, the S100A8/A9 dimerized form increases and is involved in neutrophil degranulation, cytokine production, leukocyte infiltration, and is associated with enhanced phagocytosis (56–58). We detected increased S100A8 and S100A9 protein levels in the double-tg mice as early as day 7 p.i., prior to demyelination onset, compared to control mice as assayed *via* ELISA, which correlated with increased transcripts highlighted in our scRNAseq data, suggesting S100A8 and S100A9 as potential early targets for ameliorating increased demyelination. NGAL can enhance expression of chemokines *Cxcl9* and *Cxcl10*, which in turn can increase recruitment of T cells and T cell-mediated demyelination, as well as form an MMP-9/NGAL complex to stabilize MMP-9 from degradation and enhance its function (59, 60). Therefore, selective targeting of either NGAL or downstream-related targets such as CXCL9/CXCL10 or the NGAL/MMP-9 complex, could potentially also help dampen increases in demyelination seen in response to neutrophil-enhanced demyelination.

Analysis *via* flow cytometry at the earlier timepoint of day 7 p.i., prior to the onset of severe spinal cord demyelination and clinical disease, revealed early accumulation of significantly more neutrophils within the spinal cords of the double-tg mice compared to the single-tg control mice, consistent with earlier studies using these mice (34). When looking at surface markers at day 7 p.i. on neutrophils within the spinal cords, we found increased expression for CD63, a marker of degranulation suggestive of increased effector functions. Using ImageStream flow cytometry on these samples we detected a marked increase in the overall granularity of these neutrophils in both the brain and spinal cords compared to the blood of the double-tg mice and a greater amount of CD63 staining along the perimeter of the cellular membrane. Neutrophil surface expression of CD63 has been associated with secretion of neutrophil primary granules, which in the process of degranulation are known to contain pro-inflammatory molecules, including NE and MPO (61). In accordance with this, we found increased levels of NE at day 7 p.i. in the spinal cords of double-tg mice as compared to control, which provides another early target for potential intervention in the development of demyelination. There is an increase in both CXCL1 mRNA transcripts and protein within the spinal cords of doxycycline treated double-tg mice and this correlated well with the corresponding increase in neutrophil accumulation. Whether the GFAP promoter system is more active within spinal cord astrocytes compared to astrocytes within the brains is also a possible explanation for these results and ongoing studies are exploring this possibility.

While our scRNAseq data revealed a drastic increase in the presence of neutrophils, it also showed increases in infiltrating monocytes, a general increase across all populations of microglia (MG1, MG2, Cyc. MG) and modest increases in all subsets of T cells (CD4, CD8 1, CD8 2, and Cyc. T cells). Along these lines, we have determined there is increased expression of mRNA transcripts encoding the T cell chemoattractant chemokines CXCL9 and CXCL10 within the spinal cords of JHMV-infected double-tg mice and this correlates with increased T cell infiltration, as determined by scRNAseq data and increased demyelination. Blocking CXCL10 but not CXCL9 in mice persistently infected with JHMV with established demyelination leads to a decrease in T cell infiltration into the CNS and macrophage activation that was associated with diminished white matter damage (16). Therefore, sustained neutrophil infiltration within the CNS of JHMV-infected double-tg mice may increase expression of these T cell chemoattracant chemokines in both astrocytes and microglia (6, 39). In addition, inflammatory neutrophils may directly express CXCL9 and CXCL10, which has been previously shown to impact recruitment of inflammatory T cells in transgenic mice that replicate hepatitis B (62). We and others have recently determined that microglia are important in restricting the severity of ongoing demyelination in JHMV-infected mice (38, 63). CXCL1 has been shown to alter microglia activation states *via* CXCR2 signaling (64, 65), therefore it is a possibility that chronic CXCL1 expression within the spinal cords of JHMV-infected double-tg mice could contribute to demyelination *via* impacting microglia function and impeding the ability of these cells to alter the immunologic environment and restrict white matter damage. Another intriguing possibility that we are currently exploring is how chronic CXCL1 expression impacts oligodendrocyte function within the context of persistent JHMV infection. Studies from our laboratory have shown an important role for CXCL1 signaling in impacting oligodendroglia function *via* signaling through CXCR2 (66, 67) and this raises the possibility that chronic expression of CXCL1 may ultimately negatively impact either the oligodendrocyte progenitor cell pool *via* restricting maturation to mature oligodendrocytes or affect myelin synthesis by oligodendrocytes. Collectively, findings derived from these studies add to growing evidence that innate immune responses within the CNS have important roles in regulating both neuroinflammation and demyelination.

## Data availability statement

The RNAseq data in this study have been deposited in NCBI's Gene Expression Omnibus and are accessible through GEO Series accession number GSE212852(https://www.ncbi.nlm.nih.gov/geo/query/acc.cgi?acc=GSE212852).

## Ethics statement

The animal study was reviewed and approved by UC Irvine Institutional Animal Care and Use committee.

## Author contributions

DS and AS designed and performed experiments, analyzed and interpreted data, created figures, and helped write the manuscript. GO, CS and BH helped perform experiments, interpret data and manuscript preparation. TL designed experiments, assisted with data analysis and interpretation, and helped write the manuscript. All authors contributed to the article and approved the submitted version.

## Funding

This work was supported by funding from the National Institutes of Health (NIH) grant R35NS116835, National Multiple Sclerosis Society (NMSS) Collaborative Research Center Grant CA-1607-25040 and The Ray and Tye Noorda Foundation to TEL. GMO was supported by NIH Training Grant 5T32 AI007319-33.

## Acknowledgments

The authors gratefully acknowledge the technical assistance of Cynthia Manlapaz and Kellie Fernandez.

## Conflict of interest

The authors declare that the research was conducted in the absence of any commercial or financial relationships that could be construed as a potential conflict of interest.

## Publisher’s note

All claims expressed in this article are solely those of the authors and do not necessarily represent those of their affiliated organizations, or those of the publisher, the editors and the reviewers. Any product that may be evaluated in this article, or claim that may be made by its manufacturer, is not guaranteed or endorsed by the publisher.
